# Supplementary figures and images for: iTRAQ-Based Quantitative Proteomic Analysis of Digestive Juice across the First 48 Hours of the Fifth Instar in Silkworm Larvae
Source: Int J Mol Sci. 2019 Dec 4;20(24):6113. doi: 10.3390/ijms20246113 (PMC6940845; doi:10.3390/ijms20246113)

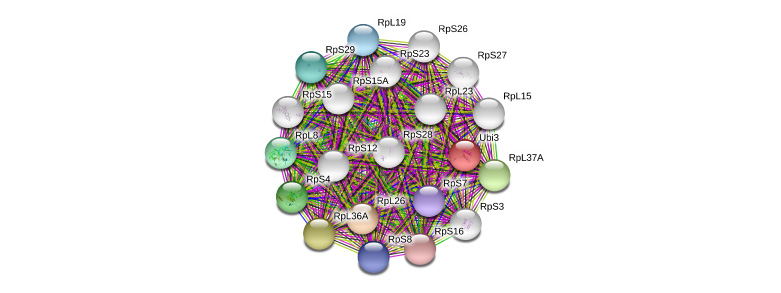

Supplement: Supplementary file 1 [file ijms-20-06113-s001.zip › supplementary files/Figure S1.jpg]
